# Gastrointestinal permeability and kidney injury risk during hyperthermia in young and older adults

**DOI:** 10.1113/EP092204

**Published:** 2024-10-17

**Authors:** Zachary J. McKenna, Whitley C. Atkins, Taysom Wallace, Caitlin P. Jarrard, Craig G. Crandall, Josh Foster

**Affiliations:** ^1^ Institute for Exercise and Environmental Medicine Texas Health Presbyterian Hospital Dallas Dallas Texas USA; ^2^ Department of Internal Medicine University of Texas Southwestern Medical Center Dallas Texas USA; ^3^ Department of Health, Exercise and Sports Sciences University of New Mexico Albuquerque New Mexico USA; ^4^ Applied Clinical Research Department University of Texas Southwestern Medical Center Dallas Texas USA; ^5^ Centre for Human and Applied Physiological Sciences King's College London London UK

**Keywords:** ageing, heat, intestinal barrier, kidney

## Abstract

We tested whether older adults, compared with young adults, exhibit greater gastrointestinal permeability and kidney injury during heat stress. Nine young (32 ± 3 years) and nine older (72 ± 3 years) participants were heated using a model of controlled hyperthermia (increasing core temperature by 2°C via a water‐perfused suit). Gastrointestinal permeability was assessed using a multi‐sugar drink test containing lactulose, sucrose and rhamnose. Blood and urine samples were assayed for markers of intestinal barrier injury [plasma intestinal fatty acid binding protein (I‐FABP), plasma lipopolysaccharide binding protein (LBP) and plasma soluble cluster of differentiation 14 (sCD14)], inflammation (serum cytokines), kidney function (plasma creatinine and cystatin C) and kidney injury [urine arithmetic product of IGFBP7 and TIMP‐2 (TIMP‐2 × IGFBP7), neutrophil gelatinase‐associated lipocalin and kidney injury molecule‐1]. The lactulose‐to‐rhamnose ratio was increased in both young and older adults (group‐wide: Δ0.11 ± 0.11), but the excretion of sucrose was increased only in older adults (Δ1.7 ± 1.5). Young and older adults showed similar increases in plasma LBP (group‐wide: Δ0.65 ± 0.89 µg/mL), but no changes were observed for I‐FABP or sCD14. Heat stress caused similar increases in plasma creatinine (group‐wide: Δ0.08 ± 0.07 mg/dL), cystatin C (group‐wide: Δ0.16 ± 0.18 mg/L) and urinary IGFBP7 × TIMP‐2 [group‐wide: Δ0.64 ± 0.95 (pg/min)^2^] in young and older adults. Thus, the level of heat stress used herein caused modest increases in gastrointestinal permeability, resulting in a mild inflammatory response in young and older adults. Furthermore, our data indicate that older adults might be more at risk for increases in gastroduodenal permeability, as evidenced by the larger increases in sucrose excretion in response to heat stress. Finally, our findings show that heat stress impairs kidney function and elevates markers of kidney injury; however, these responses are not modulated by age.

## Abstract (highlights)

- **What is the central question of this study?**
  Do older adults exhibit greater gastrointestinal permeability and heightened kidney injury risk during heat stress than young adults?
- **What is the main finding and its importance?**
  Hyperthermia increased gastrointestinal permeability, resulting in a mild inflammatory response in both young and older adults. However, older adults might be more at risk for increases in gastroduodenal permeability in response to heat stress. In addition, we show that heat stress impairs kidney function and elevates markers of kidney injury; however, these responses are not modulated by age during controlled hyperthermia. These findings serve as an important step towards improving our understanding of the risk factors that might contribute to heat‐related morbidity/mortality.

## 1 INTRODUCTION

Extreme heat events are the leading cause of death among natural disasters (Coates et al., 2014; Luber & McGeehin, 2008), and they are increasing in frequency, intensity and duration, posing a serious threat to human health (Meehl & Tebaldi, 2004; Schar et al., 2004). Individuals aged ≥65 years of age are at increased risk of morbidity and mortality during extreme heat events (Johnson et al., 2005; Knowlton et al., 2009; Linares & Diaz, 2007; Semenza et al., 1999), which is concerning when considering that the number of individuals over the age of 65 years is increasing at an accelerated pace (United States Census Bureau, 2017). For example, mortality during the 1995 heat wave in Chicago (Whitman et al., 1997) and the 2003 heat wave in France (Fouillet et al., 2006) were mostly exclusive to those aged >65 years. Determining the physiological and biochemical responses to heat stress is crucial for the development of strategies aimed at improving resilience and survival during extreme heat events.

Heat‐related illnesses are multifaceted and seem to involve hyperthermia‐evoked stress/impairment in several organ systems, including the gastrointestinal and renal systems, which have been implicated in the pathophysiology of heat stroke (Bouchama & Knochel, 2002; Bouchama et al., 2022). Hyperthermia results in vasoconstriction in the splanchnic and renal vascular beds, redistributing blood flow from these central compartments towards the skin to facilitate heat loss (Minson et al., 1998). Although these adjustments are indispensable for thermoregulation, the prolonged relative ischaemia to these organs reduces oxygen supply and is likely to cause hypoxia‐related cell damage (Hall et al., 2001).

In humans and animals, hyperthermia damages the intestinal barrier, leading to microbial translocation (Bynum et al., 1979; Gathiram et al., 1987; Leon & Helwig, 2010; Selkirk et al., 2008). Microbial translocation initiates a cascade of signalling events that can lead to systemic inflammation and eventually blood coagulation, resulting in adverse cardiovascular events and/or multiple organ failure (Bouchama et al., 2005; Huisse et al., 2008). Observational data derived from heat waves indicate that the extent of microbial translocation might be related to the severity of heat‐related health complications (Dematte et al., 1998; Huisse et al., 2008). As such, in animals pretreatment with antibiotics or anti‐bacterial antibodies prior to heat stress prevents hyperthermia‐induced mortality (Bynum et al., 1979; Gathiram et al., 1987). Despite the well‐known impact of gastrointestinal barrier integrity in the development of heat stroke (Bynum et al., 1979; Gathiram et al., 1987; Lim, 2018; Lim & Mackinnon, 2006), at present it is unknown whether age per se increases the risk of hyperthermia‐induced gastrointestinal permeability, predisposing this age group to endotoxaemia and systemic inflammation. Ageing is associated with large decreases in goblet and Paneth cell function (Sovran et al., 2019), increased microbial dysbiosis (Thevaranjan et al., 2017) and systemic inflammation (Franceschi & Campisi, 2014). Older animals also have an impaired ability to deactivate endotoxin in the liver (Jin et al., 2020). Although previous studies from our laboratory (Foster et al., 2023) and others (Lee, Russell et al., 2024; Lee, Flood et al., 2024) report elevated enterocyte damage [plasma intestinal fatty acid binding protein (I‐FABP)] with environmental heat stress in older adults, it is unknown whether this translates to measurable increases in gastrointestinal permeability.

Likewise, epidemiological data suggest that many heat‐related hospitalizations and deaths in older adults are attributable to renal complications (Bobb et al., 2014; Hopp et al., 2018; Kim et al., 2018; Lim et al., 2018; McTavish et al., 2018). Indeed, older adults might be at a greater risk for heat‐related renal complications, including an increased risk of acute kidney injury, owing to age‐related alterations in kidney structure and function (Bolignano et al., 2014; Denic et al., 2017). For example, healthy ageing (i.e., in the absence of comorbidities) is associated with a substantial reduction in the number of nephrons (Denic et al., 2017) and an increase in nephrosclerosis (Rule et al., 2010), which collectively lead to a reduction in kidney function. In addition, altered fluid regulation (Miescher & Fortney, 1989; Wolf et al., 2019) might place older adults at greater risk for dehydration, which is a known risk factor for acute kidney injury (Chapman et al., 2023). Our laboratory recently reported that older adults, but not young adults, had augmented increases in plasma creatinine and cystatin C following exposure to very hot and dry heat (McKenna et al., 2024). Those data suggest that the heightened thermal strain exhibited by older adults exposed to extreme heat might contribute to reduced renal function during heat exposure. However, to date, no study has systematically investigated the impact of ageing on renal function or markers of acute kidney injury during controlled hyperthermia where the elevation in skin and core temperatures are matched across age groups.

To address these important gaps in the literature, we tested two hypotheses: (1) older adults, compared with younger adults, exhibit greater gastrointestinal permeability during heat stress; and (2) older adults have heightened markers of kidney injury during heat stress compared with younger adults. To accomplish these aims, we used a model of controlled hyperthermia and performed comprehensive assessments of gastrointestinal permeability, microbial translocation and systemic inflammation, in addition to assessments of renal function and markers of acute kidney injury in young and older adults. The outcomes of this work will improve our understanding of the consequences of ageing on responses to heat stress.

## 2 MATERIALS AND METHODS

The study protocol and informed consent were approved by the Institutional Review Boards at the University of Texas Southwestern Medical Center and Texas Health Presbyterian Hospital Dallas (STU‐2019‐1759), and the study was performed in accordance with the principles outlined in the *Declaration of Helsinki* (NCT05816551). Data were derived from nine young adults (26–38 years of age) and nine older adults (68–78 years of age) who completed this study protocol (Table 1). Participants were recruited from the greater Dallas‐Fort Worth metropolitan area. Exclusion criteria included: (1) known heart disease or other chronic medical conditions currently requiring regular medical therapy, such as cancer, diabetes, uncontrolled hypertension or uncontrolled hypercholesterolaemia; (2) currently taking tricyclic antidepressants, loop diuretics, centrally acting calcium channel blockers or β‐blockers; (3) abnormalities detected suggestive of provocable ischaemia, undetected cardiac disease or resting left bundle branch block on screening ECG; (4) current smoker or regularly smoked within the past 3 years; (5) body mass index of ≥30 kg/m^2^; (6) known gastrointestinal issues, such as Crohn's disease or ulcerative colitis; and (7) pregnant (confirmed in young females using a urine pregnancy test).

**TABLE 1:** Baseline characteristics of participants.

Characteristic	Young (*n* = 9)	Older (*n* = 9)
Sex, *n*		
Male	6	6
Female	3	3
Medications, *n*		
Statins	0	5
Angiotensin II receptor blockers	0	1
Angiotensin converting enzyme inhibitor	0	1
α‐Blockers	0	1
Selective serotonin reuptake inhibitor	0	1
Proton pump inhibitor	1	2
Testosterone	0	1
Age, years	32 ± 3	72 ± 3
Height, cm	171 ± 10	174 ± 10
Body mass, kg	69.3 ± 16.1	75.9 ± 14.0
Body surface area, m^2^	1.80 ± 0.25	1.90 ± 0.25
Lean mass, kg	50.5 ± 9.1	48.2 ± 9.9
Body fat, %	24 ± 6	32 ± 3
Urine specific gravity, a.u.	1.007 ± 0.008	1.010 ± 0.005
Systolic blood pressure, mmHg	120 ± 10	126 ± 14
Mean arterial pressure, mmHg	91 ± 7	93 ± 10
Diastolic blood pressure, mmHg	76 ± 8	77 ± 9
Heart rate, beats/min	65 ± 9	65 ± 9
Respiration rate, breaths/min	15 ± 2	14 ± 3
End tidal CO_2_, mmHg	41 ± 5	40 ± 4

*Note*: Values are presented as *n* or the mean ± SD.

### 2.1 Preliminary testing

During preliminary testing, participants completed a medical history form and heat exposure questionnaire that was used to rule out those who were likely to be heat acclimatized. In addition, we measured their height (seca 213 stadiometer), mass (Tanita WB‐8000RW), blood pressure (Connex Pro BP 3400, Welch Allyn, NY, USA), heart rate and rhythm (via ECG) and body composition via dual‐energy X‐ray absorptiometry (Lunar iDXA, GE Healthcare, Chicago, IL, USA).

### 2.2 Study design and experimental controls

Enrolled participants completed a thermoneutral control visit and a hyperthermia trial. Participants did not use non‐steroidal anti‐inflammatory drugs for ≥1 week, antibiotics for ≥3 weeks and corticosteroids for ≥6 weeks prior to enrolment, and they did not report regular/ongoing use of supplements known to influence the intestinal barrier (i.e., probiotics, glutamine, bovine colostrum, etc.). Testing took place between 08.00 and 09.00 h after an overnight fast. Additionally, participants were asked to refrain from aerobic/resistance exercise for 24 h, alcohol for 24 h and caffeine for 8 h prior to testing. Participants were provided a food log after their first visit and were instructed to mimic their food intake on the day before their second visit. Visits were separated by ≥7 days. All testing took place in a thermoneutral laboratory (22°C ± 6°C, 41% ± 5% relative humidity). During each visit, participants provided a urine sample, and a euhydrated state was confirmed by a urine specific gravity value of ≤1.025 (Atago, Bellevue, WA, USA).

### 2.3 Control visit

On the day of the control visit, participants arrived at the laboratory, confirmed adherence to the pretest guidelines and provided a urine sample to confirm euhydration. They then ingested the multi‐sugar drink (described in detail below) dissolved in 100 mL of water for determination of resting (baseline) intestinal permeability. For the next 3 h, participants collected their urine into 3 L opaque polypropylene containers. During this time, participants remained rested (seated with minimal movement) but were free to engage with screens for work and/or entertainment. Collected urine samples were then measured for volume and mixed well before two 1.5 mL aliquots were taken and centrifuged at 20,800*g* for 15 min at 4°C. One millilitre of each supernatant was then stored at −80°C.

### 2.4 Hyperthermia trial

On the day of the hyperthermia trial, participants voided their bladder ∼1 h prior to arrival and noted the time. Upon arrival, participants confirmed adherence to the pretest guidelines and again voided their bladder into a polypropylene container to confirm euhydration and to calculate their baseline urine flow rate. In addition, two 1.5 mL aliquots were taken and centrifuged at 20,800*g* for 15 min at 4°C. One millilitre of each supernatant was then stored at −80°C. Before instrumentation, the body mass of participants was measured using a precision balance scale with  ±10 g accuracy (Mettler Toledo, OH, USA). Core temperature was measured using an orally ingestible telemetric pill (e‐Celsius performance pill, BodyCap, Caen, France) that was taken ≥1 h before the baseline period. Mean skin temperature was obtained as the weighted average of local temperatures measured via thermocouples attached to the skin surface on the chest (30%), arm (30%), anterior thigh (20%) and calf (20%). Heart rate was obtained from an ECG (GE Medical Systems, Madison, WI, USA). Blood pressure was measured by using an arm cuff, with a microphone placed over the brachial artery to detect Korotkoff sounds triggered by the ECG signal (Tango M2 Stress Test Monitor, SunTech Medical). Respiratory rate and end‐tidal carbon dioxide were measured using a nasal cannula connected to a capnograph (9004 Capnocheck Plus; Smiths Medical International, Watford, UK). Following instrumentation, participants donned a tube‐lined suit that covered the entire body except for the head, hands and feet. Participants then had 10 min of seated rest, and baseline measures were recorded.

To manipulate core and skin temperatures, participants exercised on a cycle ergometer (Lode Corival Recumbent, Amsterdam, The Netherlands) at 20 W while the temperature of the water circulating through the tube‐lined suit was increased and maintained at 50°C. Additionally, a plastic sheet was draped over the torso and arms of the participants to minimize the potential for heat loss and therefore accelerate heat gain. We chose to combine the low‐intensity exercise with heat stress for two reasons: (1) humans are unlikely to be strictly lying still and supine during periods of hot weather, but individuals must still perform activities of daily living, which increase metabolic heat generation; and (2) combining exercise with heat stress increased the total heat load (with the addition of metabolic heat production) and therefore improved the likelihood of raising core temperature by 2°C.

When the core temperature was increased by 0.5°C above baseline (∼30 min into heating), participants ingested the multi‐sugar drink dissolved in 100 mL of water, heated to core temperature, for assessment of intestinal permeability (described in detail below). Participants were heated to thermal tolerance which was defined as: (1) a core temperature increase of 2°C (young, *n* = 6; older, *n* = 6); or (2) the time at which the participant expressed that they were unable to continue or had sustained symptoms of presyncope (young, *n* = 3; older, *n* = 3). We collected data on an additional young participant, but those data were excluded and are not presented herein because their hyperthermia trial was terminated early owing to profound hypocapnia associated with the heating.

After thermal tolerance was reached, the temperature of the water perfusing the tube‐lined suit was reduced to cool the participant. After a brief period of cooling, participants towelled off and measured their nude body mass. Participants then drank ≥500 mL of water but remained rested and fasted for the remainder of the trial. Any urine produced for the 3 h following ingestion of the multi‐sugar drink was collected into 3 L opaque polypropylene containers. Collected urine samples were then measured for volume and mixed well before two 1.5 mL aliquots were taken and centrifuged at 20,800*g* for 15 min at 4°C. One millilitre of each supernatant was then stored at −80°C.

### 2.5 Blood sampling and analysis

Blood samples were collected through venipuncture of an arm vein into heparin, EDTA or serum separator Vacutainers in the seated upright position prior to heating (baseline) and at the point of thermal tolerance (end). We measured haematocrit (microcapillary technique) and haemoglobin (ABL90 Flex, Radiometer, Brønshøj, Denmark) to calculate changes in plasma volume (Dill & Costill, 1974). Blood samples were centrifuged to isolate plasma/serum, and aliquots were stored at −80°C for later analyses. We measured plasma (heparin) I‐FABP, plasma (EDTA) lipopolysaccharide binding protein (LBP) and plasma (EDTA) soluble cluster of differentiation 14 (sCD14) using ELISAs (Hycult Biotech, Uden, The Netherlands). I‐FABP, a cytosolic protein present in mature enterocytes located in the duodenum/jejunum, was used to detect intestinal cell injury. sCD14 and LBP were used as indirect markers of microbial translocation because these proteins are involved in the trafficking of lipopolysaccharide (LPS) to immune cells. In addition, to assess changes in renal function, we measured plasma (heparin) creatinine (Medica RA chemistry analyser) and cystatin C (Tosoh AIA‐360). Stored serum samples were sent to a nearby laboratory and assayed using a chemo/cytokine multiplex assay (LHC0009M ThermoFisher Scientific, Waltham, MA, USA) run on a Luminex MAGPIX platform (xMAP Technology, San Diego, CA, USA). Many samples from the multiplex assay fell below the standard curve for various analytes. In an effort to retain these samples/analytes and avoid extrapolation beyond the standard curve, we chose to analyse the raw fluorescence signal data, thus these data are presented as the fluorescence intensity (FI) (Breen et al., 2016). Additional details pertaining to the assays, including the coefficients of variation and dilution factors, can be found in Table S1.

### 2.6 Urine analysis

Stored urine samples were assayed for tissue inhibitor of metalloproteinase 2 (TIMP‐2), insulin like growth factor binding protein 7 (IGFBP7), neutrophil gelatinase‐associated lipocalin (NGAL) and kidney injury molecule‐1 (KIM‐1) via ELISAs (RayBiotech Life, Peachtree Corners, GA, USA). TIMP‐2 and IGFBP7 are cell cycle arrest proteins (Kashani et al., 2013), and NGAL is released from kidney tubular cells under stress (Schmidt‐Ott et al., 2007; Schrezenmeier et al., 2017), thus these values were used to indicate acute kidney injury. In addition, KIM‐1 was used as an additional marker of injury in the proximal tubule (Schlader et al., 2019). The arithmetic product of TIMP‐2 and IGFBP7 (TIMP‐2 × IGFBP7) was used as the primary indication for acute kidney injury risk (Endre & Pickering, 2014). Urinary biomarkers were normalized to urine flow rate.

### 2.7 Multi‐sugar drink

Intestinal permeability was assessed using a multi‐sugar drink test as described elsewhere (van Wijck et al., 2013). The drink was modified to include only 1 g of lactulose (Kristalose, Cumberland Pharmaceuticals, Nashville, TN, USA), 1 g of sucrose (107653, Sigma–Aldrich, St. Louis, MO, USA) and 0.5 g of l‐rhamnose (W373011, Sigma–Aldrich). Stored urine samples were sent to the UTSW Preclinical Pharmacology Core, and the concentration of each sugar was determined using a Sciex (Framingham, MA, USA) QTRAP 6500+ mass spectrometer coupled to a Shimadzu Nexera liquid chromatography system (LC‐MS/MS). The chromatographic conditions used a Waters (Milford, MA, USA) BEH‐Amide column (2.1 mm × 100 mm, 1.7 µm), with Solvent A being 0.1% formic acid in LC‐MS grade H_2_O, Solvent B being 0.1% formic acid in LC‐MS grade acetonitrile and a flow rate of 0.5 mL/min. The program was 0 min 97% B, 0–10 min gradient to 60% B, 10.1 min gradient to 20% B, 10.1–13 min hold 20% B, 13.1 min gradient to 97% B, and 13.1–17 min 97% B. Analytes were detected with the mass spectrometer in multiple reaction monitoring mode and negative electrospray ionization (ESI). Transitions monitored were as follows: rhamnose, [M‐Cl]^−^ 199.1/35.0; sucrose, [M‐H]^−^ 341.1/179.1; and lactulose, [M‐Cl]^−^ 377.1/161.1. ^13^C_6_Sucrose [M‐H]^−^ 347.1/181.1 was used as an internal standard. Prior to testing, the assay underwent basic validation to confirm adequate assay performance. The analytical ranges were 0.5–50 µg/mL for l‐rhamnose and 0.5–100 µg/mL for lactulose and sucrose. The assay linearity was *R* = 0.9991 for l‐rhamnose, *R* = 0.9986 for lactulose and *R* = 0.9992 for sucrose. The intra‐ and interassay precision were <11% and <19%, respectively. Briefly, the protocol was as follows: 2.5 µL of standards and quality controls (prepared in urine diluent) were mixed with 17.5 µL of urine diluent (Sigma p/n SAE0074). Twenty microlitres of sample, standards and controls was transferred to 1.5 mL microcentrifuge tubes and mixed with 180 µL of internal standard solution (acetonitrile, 0.1% formic acid with 20 µg/mL ^13^C_6_Sucrose). Samples, standards and controls were vortexed for 5 s and centrifuged at 16,100*g* at 4°C for 5 min. One hundred and eighty microlitres of samples, standards and control was transferred to a centrifugal filter plate and centrifuged at 550*g* at 4°C for 2 min, moved into a 96‐well sample plate, and run on the LC‐MS/MS. Peaks were integrated and peak areas determined using Multiquant v.3.0.3 Software (Sciex). Quantification was based on comparisons against the calibration curves established for each sugar in the urine diluent.

The urinary recovery of each ingested sugar (lactulose, sucrose and rhamnose) was determined by multiplying the measured concentration of each sugar by the total volume of urine collected and dividing by the dose administered. Given that lactulose is degraded in the colon, we used the ratio of urinary lactulose to rhamnose (L/R) to determine small intestinal barrier permeability. Sucrose is broken down rapidly in the duodenum, thus we used the urinary excretion of sucrose to assess gastroduodenal permeability. If lactulose or sucrose was not detected in the sample, the L/R ratio or sucrose excretion was assumed to be zero.

### 2.8 Data acquisition and calculations

Skin temperature and heart rate were sampled at 250 Hz (Biopac MP150, Santa Barbara, CA, USA), converted into 1 min averages, and taken at 5 min intervals for data analysis. Core temperature measured with the telemetric pill was sampled every 30 s and is reported at 5 min intervals. Blood pressure was measured at rest and every 15 min during heating, for safety monitoring reasons. Whole‐body sweat loss (WBSL) was calculated as the difference in nude body mass between pre‐ and post‐heating, after correcting for the volume of fluid contained in the multi‐sugar drink. Body surface area was calculated using the equation defined by Du Bois and Du Bois (1989). Urine flow rate was calculated by dividing the volume of urine produced by the total time of collection. We estimated glomerular filtration rate using the 2021 Chronic Kidney Disease Epidemiology Collaboration (CKD‐EPI) creatinine–cystatin C equation, adjusting for body surface area (Inker et al., 2021).

### 2.9 Statistical analyses

A power calculation was performed (G*Power v.3.1.9) using an estimated effect size (partial η^2 ^= 0.06) of the within (trial: control vs. hyperthermia) between (group: young vs. older) interaction on the L/R ratio. With an α level of 0.05, power of 0.80 (1 − β) and assuming a moderate correlation (*r* = 0.75) among repeated measures, we estimated that 18 participants (nine young and nine older) would be required to detect differences in change in (Δ) L/R between young and older participants. Before statistical analyses, data were assessed to confirm that they met model assumptions (i.e., normality and equality of variance), and the data were inspected for influential data points. Non‐normally distributed data were transformed using a logarithmic (base 10) transformation. When a logarithmic transformation was required, measurement units were adjusted (i.e., converting micrograms to nanograms) to avoid logarithmic transformation of values less than one. We analysed data using linear mixed effect models, with main effects of time or trial (within factor) and group (between factor; older vs. young) or Student's unpaired *t*‐tests, as appropriate. Statistical analyses were performed in Prism v.9.4 (GraphPad Software, La Jolla, CA, USA). Data in the text, tables and figures are presented as the mean ± SD. Statistical significance was set a priori to *P* < 0.05.

## 3 RESULTS

### 3.1 Physiological responses to hyperthermia

**FIGURE 1:**
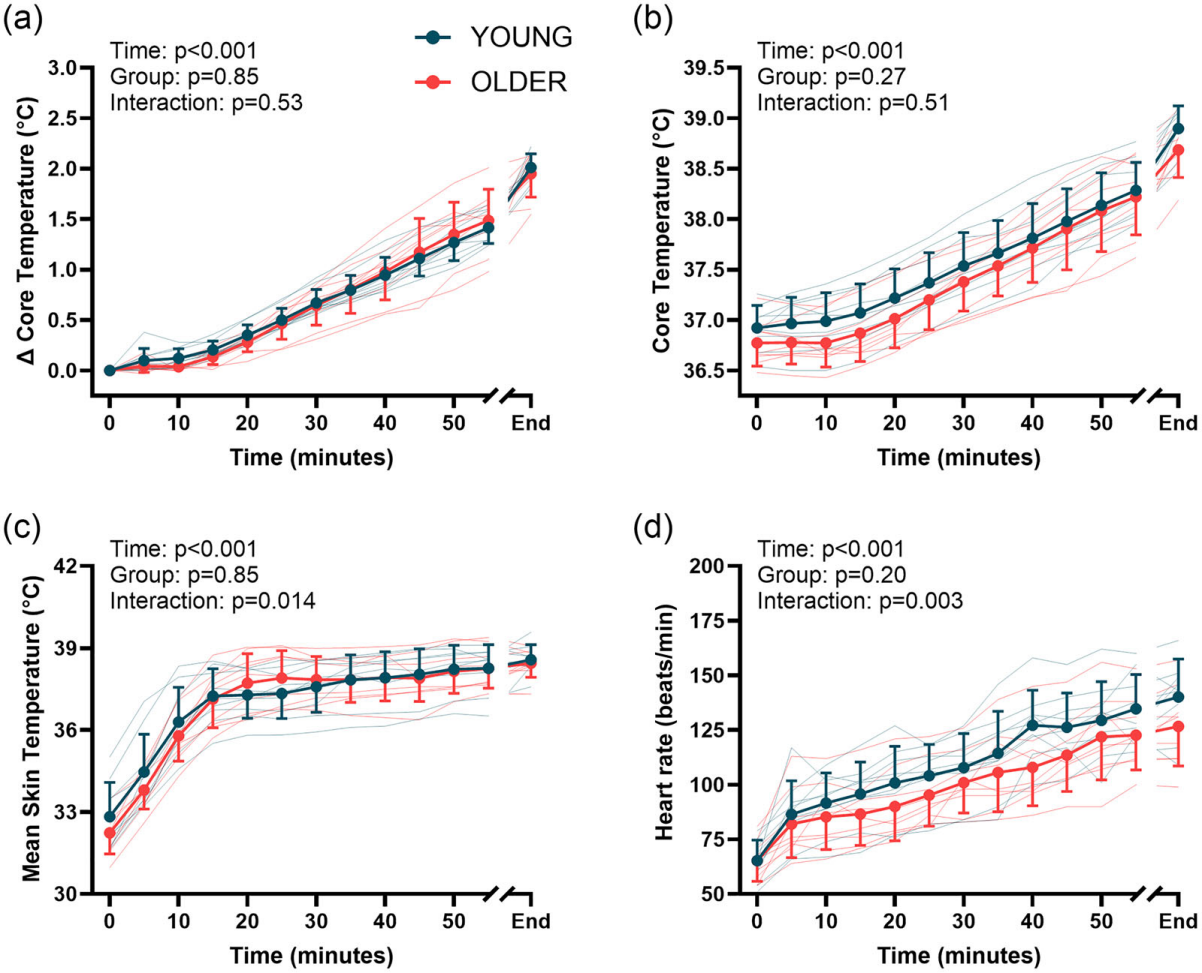
Physiological responses during the hyperthermia trial. Change in core temperature (a), absolute core temperature (b), mean skin temperature (c) and heart rate (d) responses are shown in young (*n* = 9) and older participants (*n* = 9). Data were compared using linear mixed‐effects models, with main effects of time and group.

Total heating time was 72 ± 4 min in the young cohort and 67 ± 10 in the older cohort (*P* = 0.19), with a peak temperature increase of 2.01°C ± 0.14°C for young participants and 1.95°C ± 0.23°C for older participants (*P* = 0.50; Figure 1a). The peak core temperature was 38.90°C ± 0.22°C in young participants and 38.69°C ± 0.27°C in older participants (*P* = 0.09; Figure 1b). Complete core temperature, heart rate and skin temperature results from the hyperthermia trial are shown in Figure 1. Whole‐body sweat loss was greater in young participants compared with the older participants (1.32 ± 0.54 vs. 0.73 ± 0.57 L, respectively; *P* = 0.04). The change in plasma volume was not different between young and older participants (−4.1% ± 3.9% vs. −7.2% ± 6.0%, respectively; *P* = 0.22).

### 3.2 Markers of gastrointestinal barrier function

**FIGURE 2:**
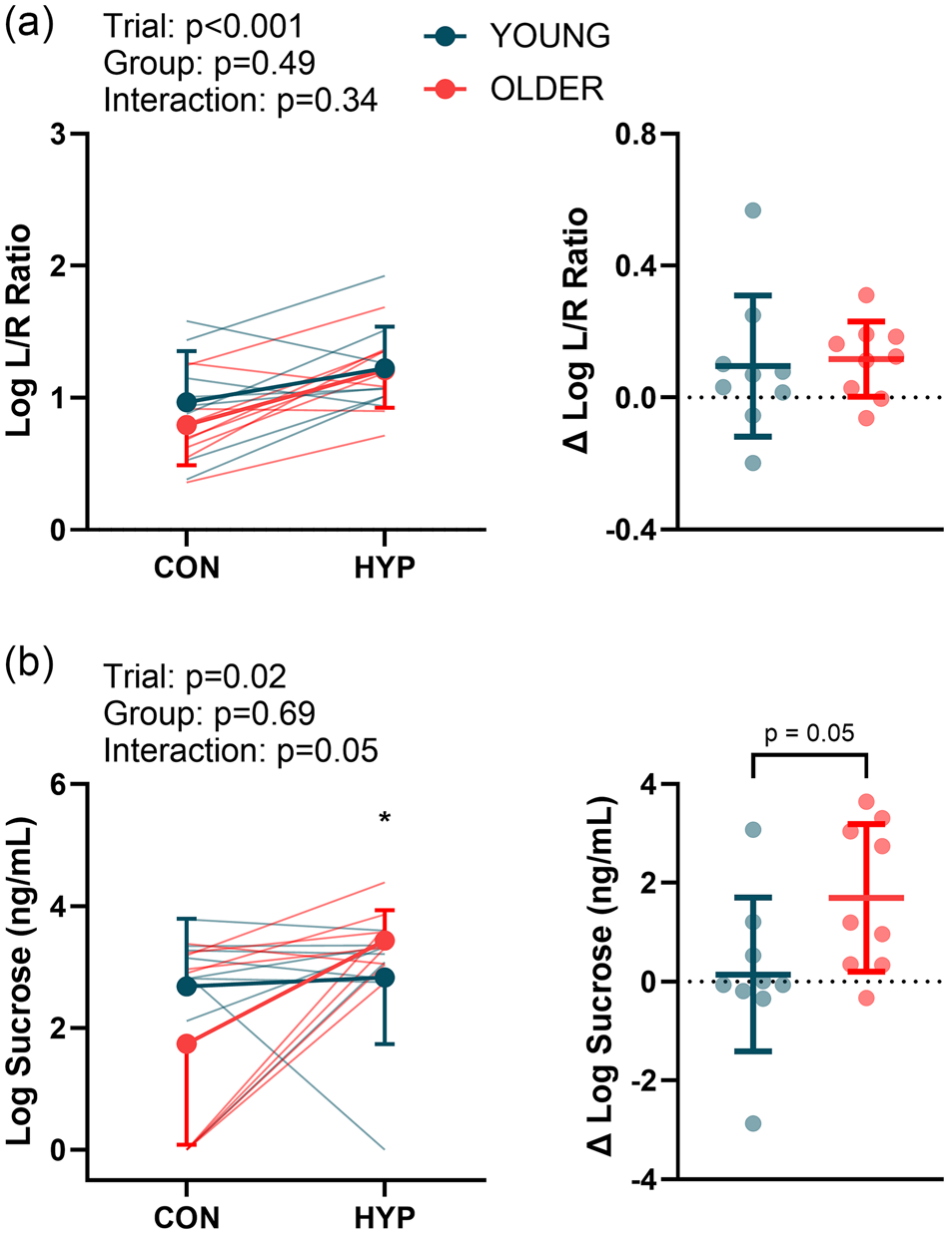
Intestinal permeability was assessed during control (CON) and hyperthermia (HYP) trials in young (*n* = 9) and older (*n* = 9) participants. (a) The lactulose‐to‐rhamnose (L/R) ratio shows small intestinal permeability. (b) Sucrose excretion shows gastroduodenal permeability. The graphs on the right of each panel show the change from the control trial. Non‐normally distributed data were logarithmically (base 10) transformed prior to analyses. Data were compared using linear mixed effects models, with main effects of trial and group. *Significant difference (*P* = 0.004) between CON and HYP in older adults.

**FIGURE 3:**
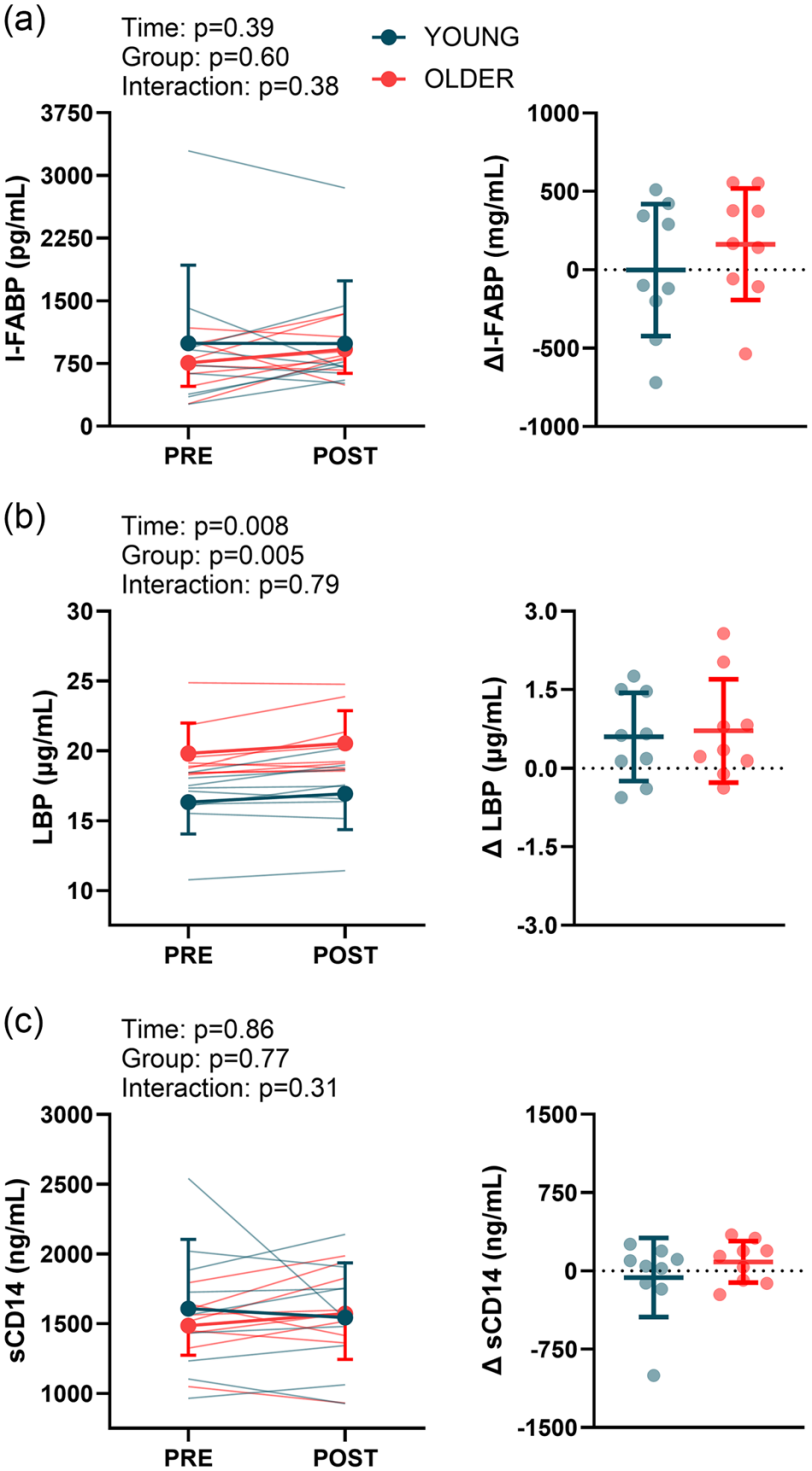
Markers of intestinal cell injury and microbial translocation during the hyperthermia trial in young (*n* = 9) and older (*n* = 9) participants. Plasma concentrations of intestinal fatty acid binding protein (I‐FABP; a), lipopolysaccharide binding protein (LBP; b) and soluble cluster of differentiation 14 (sCD14; c) are shown pre‐ and post‐heating. The graphs on the right of each panel show the pre‐ to post‐heating changes. Data were compared using linear mixed effects models, with main effects of time and group.

The L/R ratio (log_10_‐transformed) was greater than the control following the hyperthermia trial in both young (0.97 ± 0.39 vs. 1.22 ± 0.32) and older (0.79 ± 0.30 vs. 1.21 ± 0.29) adults (main effect of trial, *P *< 0.001), but there was no main effect of group or interaction (Figure 2a). There was an increase in the urinary excretion of sucrose (log_10_‐transformed) from control in older (1.74 ± 1.66 vs. 3.44 ± 0.50 ng/mL; *P* = 0.004), but not young (2.68 ± 1.11 vs. 2.83 ± 1.10 ng/mL) adults (interaction, *P* = 0.05; Figure 2b). There was not a main effect of time, group or interaction for I‐FABP (young, from 991 ± 935 to 990 ± 749 pg/mL; older, from 757 ± 282 to 920 ± 287 pg/mL) or sCD14 (young, from 1607 ± 497 to 1545 ± 392 ng/mL; older, from 1485 ± 212 to 1573 ± 328 ng/mL) (Figure 3a,b). There was a pre‐ to post‐heating increase in LBP in both groups (young, from 16.35 ± 2.30 to 16.94 ± 2.57 µg/mL; older, from 19.81 ± 2.18 to 20.52 ± 2.34 µg/mL) (main effect of time, *P* = 0.008). Older adults had higher LBP compared with young adults (main effect of age, *P* = 0.005), but there was no time by group interaction (Figure 3c).

### 3.3 Circulating markers of inflammation

**FIGURE 4:**
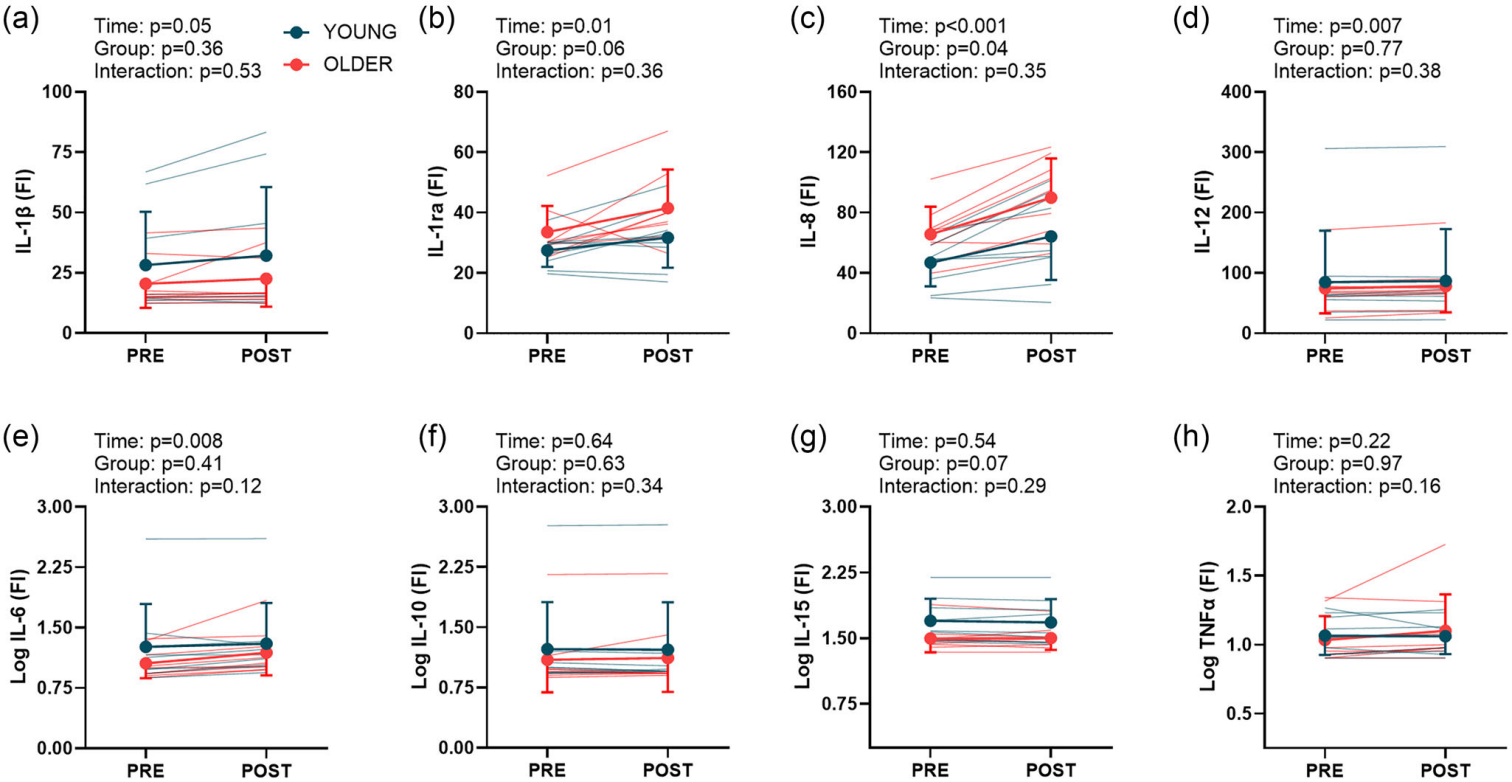
Results of the serum cytokine panel during the hyperthermia trial in young (*n* = 9) and older (*n* = 9) participants. The fluorescence intensity of interleukin (IL)‐1β (IL‐1β; a), IL‐1ra (b), IL‐8 (c), IL‐12 (d), IL‐6 (e), IL‐10 (f), IL‐15 (g) and tumour necrosis factor‐α (TNFα; h) are shown. Non‐normally distributed data were logarithmically (base 10) transformed prior to analyses. Data were compared using linear mixed effects models, with main effects of time and group.

Results from the cytokine panel are shown in Figure 4, and the mean ± SD values are listed in Table S2. There were pre‐ to post‐heating increases in the fluorescence intensity for IL‐1β, IL‐1ra, IL‐6, IL‐8 and IL‐12 (main effect of time, *P* ≤ 0.05 for all indices). In addition, older adults had greater fluorescence intensity for IL‐8 (main effect of age, *P* = 0.04). However, we did not detect a significant time by group interaction for any marker of inflammation (Figure 4).

### 3.4 Markers of kidney function and injury

**FIGURE 5:**
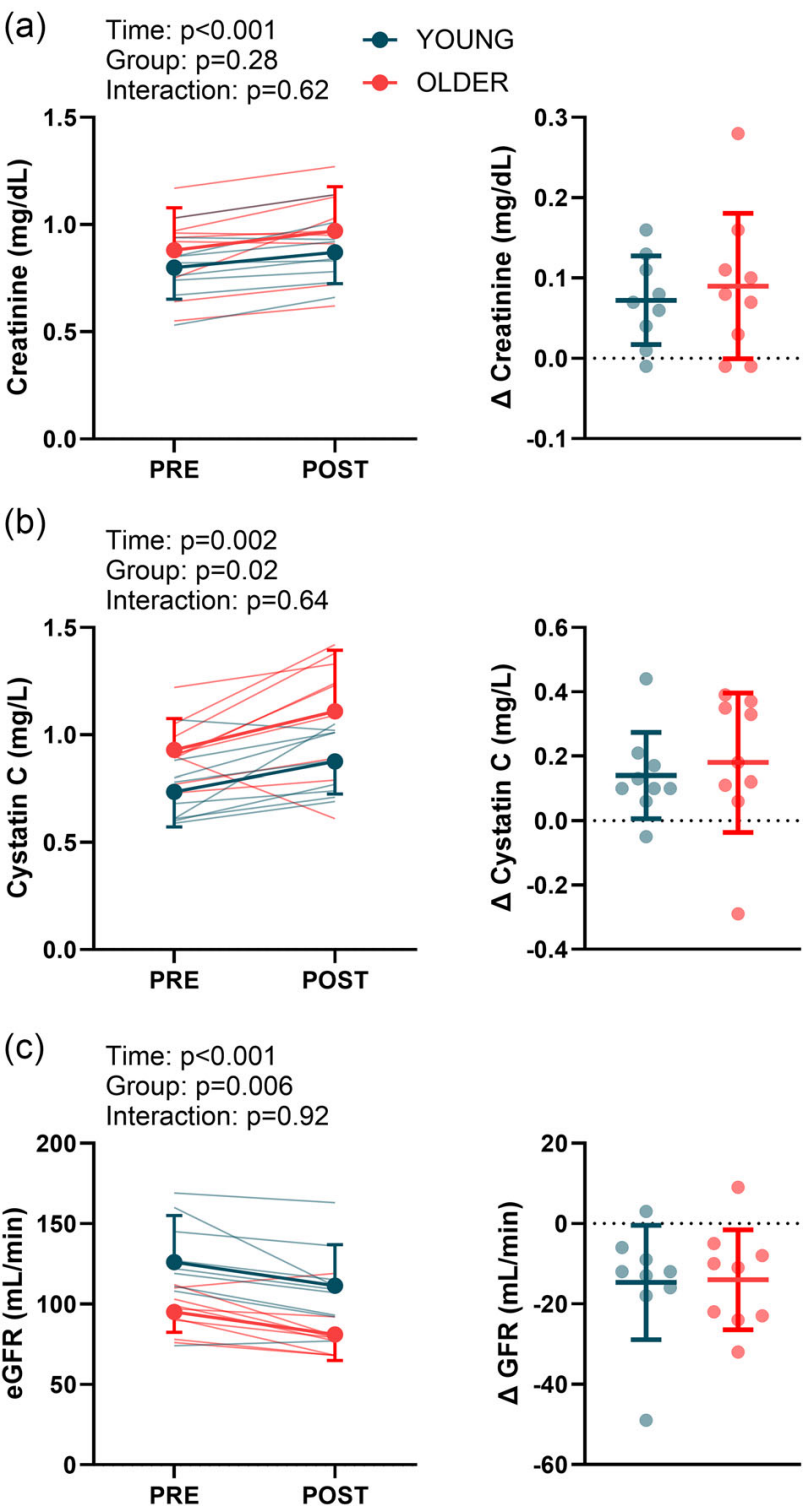
Markers of kidney function during the hyperthermia trial in young (*n* = 9) and older (*n* = 9) participants. Plasma creatinine (a), plasma cystatin C (b) and estimated glomerular filtration rate (eGFR; c) are shown pre‐ and post‐heating. The graphs on the right of each panel show the pre‐ to post‐heating changes. Data were compared using linear mixed effects models, with main effects of time and group.

**FIGURE 6:**
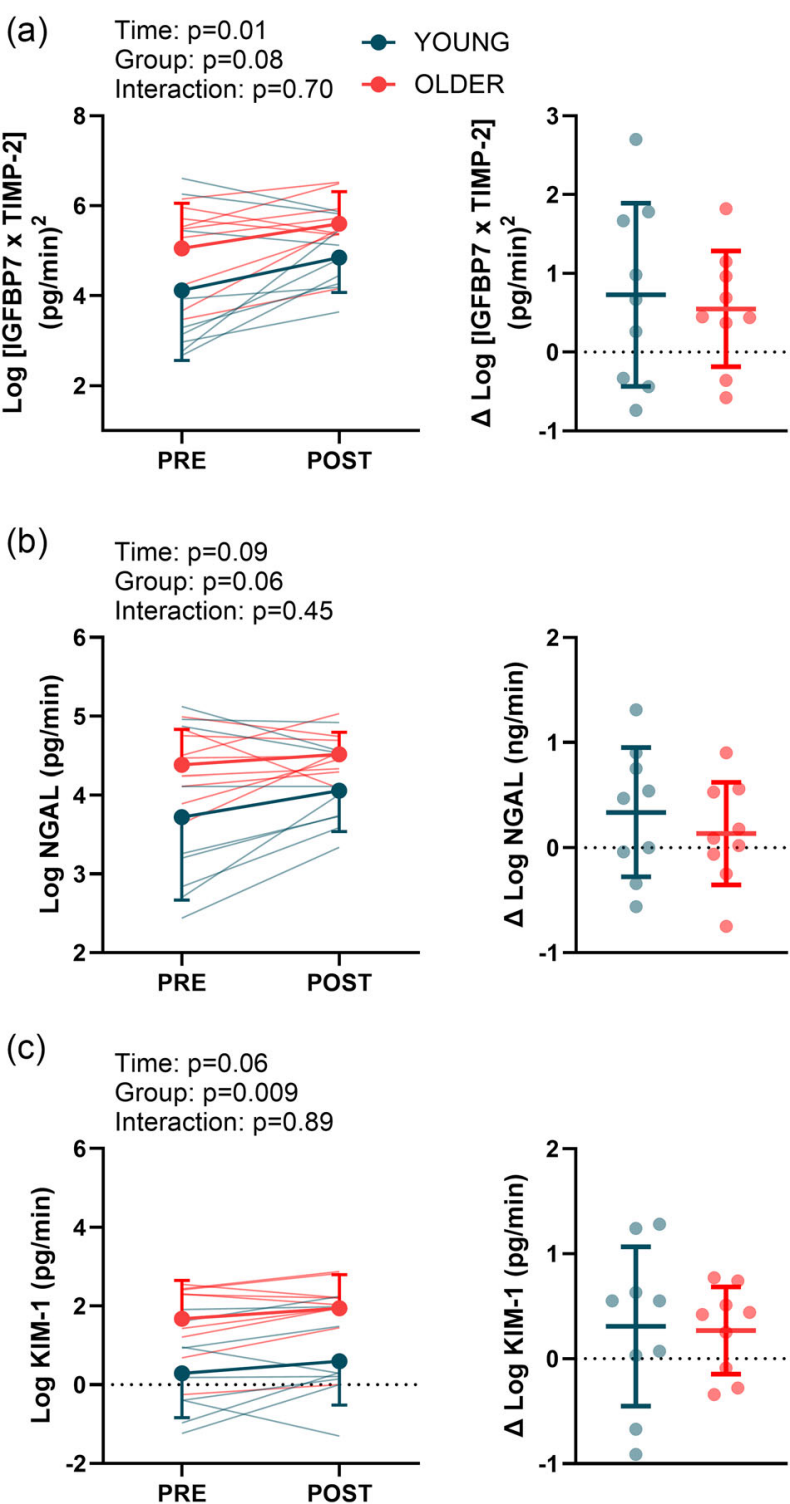
Markers of acute kidney injury during the hyperthermia trial in young (*n* = 9) and older (*n* = 9) participants. Urinary concentrations of arithmetic product of IGFBP7 and TIMP‐2 (TIMP‐2 × IGFBP7; a), neutrophil gelatinase‐associated lipocalin (NGAL; b) and kidney injury molecule‐1 (KIM‐1; c) are shown pre‐ and post‐heating. The graphs on the right of each panel show the pre‐ to post‐heating changes. These markers have been normalized to urine flow rate. Non‐normally distributed data were logarithmically (base 10) transformed prior to analyses. Data were compared using linear mixed effects models, with main effects of time and group.

There was a main effect of group (*P* = 0.03), but not time (*P* = 0.06) or interaction (*P* = 0.13) for urine flow rate. Baseline urine flow rate was 4.23 ± 3.40 mL/min in young participants and 1.70 ± 1.49 mL/min in older participants. Post‐heating urine flow rate was 2.41 ± 1.08 mL/min in the young participants and 1.49 ± 0.48 mL/min in older participants. Creatinine and cystatin C were greater following the hyperthermia trial compared with control in both young and older adults, but there was no main effect of group or interaction (Figure 5a,b). Likewise, estimated glomerular filtration rate was reduced by −15 ± 14 mL/min in older participants and by −14 ± 12 mL/min in young participants (Figure 5c). There was a main effect of time for IGFBP7 × TIMP‐2 (Figure 6a; *P* = 0.01), but no main effect of group (*P* = 0.08) or interaction (*P* = 0.70). There were no main effects of time, group or interaction for NGAL or KIM‐1 (Figure 6b,c).

## 4 DISCUSSION

In this study, we tested the hypothesis that older adults, compared with young adults, exhibit greater gastrointestinal permeability and kidney injury during heat stress. Accordingly, we used a model of controlled hyperthermia (i.e., similar levels of core temperature elevation) and performed comprehensive assessments of gastrointestinal permeability, microbial translocation and systemic inflammation, in addition to assessments of kidney function and acute kidney injury in young and older adults. Our key findings are as follows: (1) hyperthermia increased small intestinal permeability (L/R ratio) in both young and older adults, but gastroduodenal permeability (excretion of sucrose) was increased in older but not young adults; (2) hyperthermia increased LBP but had no effect on circulating I‐FABP or sCD14; (3) several circulating inflammatory cytokines, such as IL‐1β, IL‐1ra, IL‐6, IL‐8 and IL‐12, were increased with hyperthermia; (4) hyperthermia increased circulating creatinine and cystatin C, indicating reduced kidney function; and (5) some (IGFBP7 × TIMP‐2), but not all (NGAL and KIM‐1), markers of acute kidney injury were elevated in both young and older adults following heat stress. Taken together, these findings suggest that the level of hyperthermia used herein (Δ2°C) causes modest increases in intestinal permeability, resulting in a mild inflammatory response in both young and older adults. Furthermore, our data indicate that older adults might be more at risk for increases in gastroduodenal permeability, as evidenced by the greater increases in sucrose excretion in response to heat stress. Finally, our findings show that heat stress causes an acute decline in renal function and elevated markers of acute kidney injury risk; however, these responses do not appear to be modulated by age. These findings highlight several unique responses to heat stress in both young and older adults that were previously unknown. Understanding these responses is crucial when considering the risk factors that contribute to heat‐related health complications and might eventually aid in the development of strategies aimed at improving resilience to heat stress.

Heat stress, such as that observed during physiologically relevant increases in core temperature (i.e., from 37°C to 41°C), is known to increase intestinal permeability in vitro by disrupting tight junction protein structures and damaging intestinal epithelial cells (Dokladny et al., 2006, 2016). Older adults, having recognized impairments in thermoregulation, are more likely to develop hyperthermia during environmental heat stress and therefore might be more at risk for heat‐related gastrointestinal complications (McKenna et al., 2023). Indeed, our previous work and that of others have shown that older adults, but not young adults, had increased I‐FABP, a marker of intestinal cell damage, following ambient heat stress when core temperatures were higher in older individuals (Foster et al., 2023; Lee, Russell et al., 2024; Lee, Flood et al., 2024). However, in addition to impaired thermoregulation, ageing alone is associated with altered intestinal barrier function (Pentinmikko et al., 2019; Sovran et al., 2019; Thevaranjan et al., 2017), decreased microbial neutralization (Jin et al., 2020) and lower resting splanchnic blood flow (Minson et al., 1998). For these reasons and in an attempt to isolate the primary effects of ageing, we used a model of controlled hyperthermia whereby young and older participants were exposed to similar levels of thermal strain (i.e., matched increases in core temperature). Using this model, we demonstrated that heat stress increases small intestinal permeability (L/R ratio) in both young and older adults. Interestingly, our findings show that older, but not young, adults have heat‐induced increases in sucrose excretion, suggesting increased gastroduodenal permeability. Although the exact mechanisms underlying this finding are not known, this finding suggests that older adults are more susceptible to heat‐induced upper gastrointestinal barrier damage. Nonetheless, the impact or relevance of this response on microbial translocation and subsequent inflammation is unclear.

The pre‐ to post‐heating increase in LBP supports the parallel increases in small intestinal permeability (L/R). Both LBP and sCD14 are acute phase proteins involved in the trafficking of LPS to immune cells, thus increases in these markers have been used as surrogates for LPS translocation. Although we found small pre‐ to post‐heating increases in LBP, we did not see changes in sCD14. These findings are similar to a previous study that reported increases in LBP but not sCD14 following sauna‐induced dehydration (3% body mass loss, tympanic temperature 38.6°C) (Roca Rubio et al., 2021). The reasons underlying differential LBP and sCD14 responses are not clear, but they might be attributable to differences in regulatory mechanisms or analyte sensitivity. For example, given that we found only mild increases in permeability, it might be that LBP is a slightly more sensitive marker of LPS translocation compared with sCD14. Alternatively, LBP is produced and secreted by hepatocytes and intestinal epithelial cells, whereas sCD14 is produced primarily by immune cells (Maliszewski, 1991; Schumann et al., 1990; Vreugdenhil et al., 1999; Wright et al., 1990). Therefore, heat stress might have caused increases in LBP via production and release locally in the gut (via intestinal epithelial cells) or from the liver, but not sufficient to cause immune activation or turnover leading to changes in sCD14.

Surprisingly, we did not observe pre‐ to post‐heating increases in I‐FABP. We hypothesized that this model of heat stress would lead to enterocyte damage secondary to reductions in intestinal blood flow and perhaps direct thermal injury (Dokladny et al., 2006; Minson et al., 1998; van Wijck et al., 2012). Although our protocol resulted in a near 2°C increase in core temperature, probably resulting in reductions in splanchnic perfusion (Minson et al., 1998; Rowell et al., 1970, 1971), our results suggest that this model of heat stress did not cause intestinal cell damage. Our findings are supported by Roca Rubio et al. (2021), who found that sauna‐induced dehydration (15 min periods at 70°C interspersed with 10 min cooling periods) did not increase I‐FABP in young adults. However, these findings are in contrast to our previous study and others, which reported increases in I‐FABP in older adults during ambient heat exposure (Foster et al., 2023; Lee, Russell et al., 2024; Lee, Flood et al., 2024). One important consideration is that the total heating time used herein (∼1 h) was shorter than our previous protocol (3 h), which might explain these different results. In support of this, Lee, Flood et al., et al. (2024) recently reported increases in I‐FABP in older adults, but not young adults, following ∼3 h of exertional heat stress. Thus, it seems that there might be an effect of the duration of heating that is required to cause intestinal cell damage. Considering that we took participants to their thermal tolerance, it is not plausible that individuals could sustain such severe elevations in core temperature (Δ2°C) for an additional 2 h. Thus, to address this question in the future, we suggest using alternative methods of heating, such as ambient heating, which leads to more gradual increases in core temperature over time. An advantage of an ambient heating approach is that it is more reflective of what occurs in real extreme heat conditions. However, one key disadvantage is that it is difficult to isolate the primary effects of ageing on the responses to heat stress, given that older adults are more likely to have larger core temperature responses (Kenny et al., 2017; McKenna et al., 2023; Meade et al., 2023; Miescher & Fortney, 1989).

There were small pre‐ to post‐heating increases in circulating inflammatory cytokines. Before interpreting these cytokine responses, we want to highlight that we chose to report the fluorescence intensity (FI) of the cytokines, as opposed to the estimated concentrations. This was done to retain a full data set of samples, including those that fell below the detection range of the assay, and to avoid extrapolation, because this has the potential to introduce bias (Breen et al., 2016). For this reason, we will limit our interpretation to the impact of heat and age on the pre‐ to post‐heating change of these cytokines. The increases in IL‐1β, IL‐1ra, IL‐6, IL‐8 and IL‐12 support that the heat stress resulted in a mild inflammatory response, perhaps attributable to microbial translocation or other involved mechanisms. For example, IL‐1β and IL‐6 are classically known as pro‐inflammatory cytokines that play important roles in the immune response to LPS via interactions with Toll‐like receptor‐4 (Ducharme et al., 2022). Thus, taken together with the observed increases in LBP, our data suggest that heat stress might have resulted in a small amount of LPS translocation, although it should be noted that the increases in both circulating cytokines and LBP were small, hence this response was modest at best.

We found that heat stress increased plasma creatinine and cystatin C, in addition to urinary IGFBP7 × TIMP‐2, collectively indicating a potential risk for acute kidney injury. Creatinine and cystatin C are kidney function biomarkers that are often used in clinical settings to estimate glomerular filtration rates. We recently reported that older, but not young, adults had augmented increases in plasma creatinine and cystatin C following exposure to very hot and dry heat (McKenna et al., 2024), probably attributable to the heightened thermal strain exhibited by older adults exposed to extreme heat (McKenna et al., 2023). The findings reported herein support that rationale, because we noted similar responses between young and older during controlled (matched) hyperthermia. Likewise, the increase in IGFBP7 × TIMP‐2 builds upon those previous findings and indicates that these urinary biomarkers reflect acute changes in commonly measured blood‐based biomarkers, such as creatinine and cystatin C. Given that dehydration is an important risk factor for acute kidney injury (Chapman et al., 2020), it is important to highlight that the lack of difference in kidney markers between the young and older groups persisted despite different levels of fluid loss (−1.81% ± 0.83% for young vs. −0.86% ± 0.68% for older, *P* = 0.009). It is unclear whether these responses would be exacerbated in older adults during heat‐wave‐like conditions given the concern that older adults are less likely to maintain hydration ad libitum owing to a variety of factors (Begg, 2017; Phillips et al., 1993).

There are several methodological considerations that should be noted. Heating with the water‐perfused suit leads to faster elevations in skin and core temperatures than are typically observed during ambient heat exposure. Accordingly, although the present protocol had high internal validity, allowing for tight control of core temperature, it lacked some ecological relevance. Along these same lines, we compared young and older adults during controlled hyperthermia, where increases in core temperature were similar between groups. This was done to isolate the effect of primary ageing on the gastrointestinal and renal responses to hyperthermia. Given that older adults exhibit greater core temperature elevations during ambient heat exposure compared with young adults, ambient exposures have poor utility if isolating the direct impact of ageing (McKenna et al., 2023). However, given that thermal strain is likely to be greater in older individuals during such ambient exposures, our data suggest that this population might be more likely to suffer from adverse gastrointestinal and renal complications. Next, we show cytokine data as fluorescence intensity rather than concentration, which limits our ability to understand the full immune/inflammatory response to heat stress. However, this does not affect the ability to interpret the relative changes in each cytokine. In addition, we corrected the markers of acute kidney injury to urine flow rate. Although this approach improves the interpretation by adjusting for potential differences in hydration (Middleton et al., 2016), it is recognized that older adults can have higher post‐voiding residual volumes (Madersbacher et al., 1998). Future studies investigating age differences in markers of acute kidney injury should consider implementing corrections using other hydration metrics, such as osmolality, or using more invasive procedures (i.e., urinary catheterization). Finally, although we included a balanced number of young and older females, we lack adequate sample size to test statistically for interactions between sex, heat and age group.

In conclusion, we show that hyperthermia results in increased gastrointestinal permeability that might cause some microbial translocation and a mild inflammatory response in both young and older adults. Likewise, our findings show that heat stress increases blood‐based kidney function biomarkers and urine‐based markers of acute kidney injury risk, but these responses are not different between young and older adults during matched hyperthermia. However, our data suggest that older adults, compared with young adults, might be more at risk for increases in gastroduodenal permeability. Given that gastrointestinal barrier dysfunction and systemic inflammation are implicated in the pathophysiology of heat‐related illnesses (Bouchama & Knochel, 2002; Bouchama et al., 2022), these findings serve as an important step towards improving our understanding of the risk factors that might contribute to heat‐related morbidity and mortality.

## AUTHOR CONTRIBUTIONS

Zachary J. McKenna, Josh Foster and Craig G. Crandall contributed to conceptualization and design. Zachary J. McKenna, Whitley C. Atkins, Taysom Wallace, Caitlin P. Jarrard and Josh Foster were responsible for data collection and data analysis. Zachary J. McKenna, Craig G. Crandall and Josh Foster were responsible for the interpretation and drafting of the article. All authors reviewed the article and provided critical feedback. All authors approved the final version of the manuscript and agree to be accountable for all aspects of the work in ensuring that questions related to the accuracy or integrity of any part of the work are appropriately investigated and resolved. All persons designated as authors qualify for authorship, and all those who qualify for authorship are listed.

## CONFLICT OF INTEREST

None declared.

## Supporting information



Supporting Information

## Data Availability

The data that support the findings of this study are available from the corresponding author upon reasonable request.
