# Supplementary material for: Gastrointestinal permeability and kidney injury risk during hyperthermia in young and older adults
Source: Exp Physiol. 2024 Oct 17;110(1):79–92. doi: 10.1113/EP092204 (PMC11689130; doi:10.1113/EP092204)
Supplement: Supplementary file 1 — Supporting Information [file EPH-110-79-s001.docx]

**SUPPLEMENTAL MATERIAL**

| **Table S1**: Assay product numbers, coefficient of variations, and dilutions. | | | |
| --- | --- | --- | --- |
| **Analyte** | **Manufacturer, Catalog #** | **CV** | **Dilution** |
| I-FABP | Hycult Biotech, HK406 | 7% | 2x |
| LBP | Hycult Biotech, HK315 | 3% | 1000x |
| sCD14 | Hycult Biotech, HK320 | 4% | 90x |
| IL-1ra | ThermoFisher Scientific, LHC0009M | 13% | 2x |
| IL-1β | ThermoFisher Scientific, LHC0009M | 6% | 2x |
| IL-6 | ThermoFisher Scientific, LHC0009M | 8% | 2x |
| IL-8 | ThermoFisher Scientific, LHC0009M | 5% | 2x |
| IL-10 | ThermoFisher Scientific, LHC0009M | 7% | 2x |
| IL-12 | ThermoFisher Scientific, LHC0009M | 11% | 2x |
| IL-15 | ThermoFisher Scientific, LHC0009M | 8% | 2x |
| TNF-α | ThermoFisher Scientific, LHC0009M | 6% | 2x |
| KIM-1 | RayBiotech Life, ELH-TIM-1 | 11% | 3X |
| IGFBP7 | RayBiotech Life, ELH-IGFBPRP1 | 14% | 20X |
| TIMP-2 | RayBiotech Life, ELH-TIMP2 | 5% | 50X |
| NGAL | RayBiotech Life, ELH-Lipocalin2 | 3% | 20X |

| **Table S2:** Results of the serum cytokine panel during the hyperthermia trial in young (n=9) and older (n=9) participants. | | | |
| --- | --- | --- | --- |
| **Variable** | **Young** | **Older** | **P value** |
| **IL-1β (FI)**  Pre  Post | 28 ± 22  32 ± 28 | 20 ± 10  23 ± 12 | Time: p=0.05  Group: p=0.36  Interaction: p=0.53 |
| **IL-1ra (FI)**  Pre  Post | 28 ± 6  32 ± 10 | 34 ± 9  41 ± 13 | Time: p=0.01  Group: p=0.06  Interaction: p=0.36 |
| **Log IL-6 (FI)**  Pre  Post | 1.26 ± 0.53  1.30 ± 0.51 | 1.06 ± 0.19  1.19 ± 0.28 | Time: p=0.008  Group: p=0.41  Interaction: p=0.12 |
| **IL-8 (FI)**  Pre  Post | 47 ± 16  64 ± 29 | 66 ± 18  90 ± 26 | Time: p<0.001  Group: p=0.04  Interaction: p=0.35 |
| **Log IL-10 (FI)**  Pre  Post | 1.23 ± 0.59  1.22 ± 0.59 | 1.10 ± 0.41  1.12 ± 0.42 | Time: p=0.64  Group: p=0.63  Interaction: p=0.34 |
| **IL-12 (FI)**  Pre  Post | 85 ± 85  87 ± 86 | 74 ± 41  78 ± 43 | Time: p=0.007  Group: p=0.77  Interaction: p=0.38 |
| **Log IL-15 (FI)**  Pre  Post | 1.70 ± 0.25  1.68 ± 0.27 | 1.50 ± 0.16  1.50 ± 0.14 | Time: p=0.54  Group: p=0.07  Interaction: p=0.29 |
| **Log TNFα (FI)**  Pre  Post | 1.07 ± 0.14  1.06 ± 0.13 | 1.03 ± 0.17  1.10 ± 0.26 | Time: p=0.22  Group: p=0.97  Interaction: p=0.16 |
| Units are shown as fluorescence intensity. Non-normally distributed data were log (base 10) transformed. Data were compared using linear mixed effects models with main effects of time and group. | | | |
